# Different Knots, Same Outcome: Evaluating the Role of Surgical Technique on Biliary Anastomotic Strictures After Liver Transplantation

**DOI:** 10.1002/wjs.70288

**Published:** 2026-02-27

**Authors:** Samith Minu Alwis, Robert Torode, Michael Anthony Fink, Ruelan Furtado, Eunice Lee, Graham Starkey, Robert Jones, Marcos Vinicius Perini

**Affiliations:** ^1^ Department of Surgery (Austin Health) The University of Melbourne Melbourne Victoria Australia; ^2^ Victorian Liver Transplant Unit Austin Health Melbourne Victoria Australia

**Keywords:** biliary anastomotic strictures, graft survival, liver transplantation, surgical technique

## Abstract

**Background:**

Biliary anastomotic strictures (BAS) after liver transplant (LT) are a significant contributor to post‐transplant morbidity. Although surgical technique has been proposed as a risk factor, accurate evaluation of technique has been limited by inherent bias in conventional definitions for BAS. This study aimed to evaluate the incidence of clinically significant BAS (csBAS) with absorbable suture material and variable anastomotic suture technique in patients undergoing LT with duct‐to‐duct (DD) anastomosis.

**Methods:**

A retrospective medical record review was conducted of adult patients undergoing LT at a single center between January 1st, 2000 and December 31st, 2023. Suture technique included continuous or interrupted alone, or a combined technique (continuous to posterior wall, interrupted anteriorly), while suture material was either absorbable or non‐absorbable suture. Primary endpoint was the incidence of csBAS using a previously introduced surrogate marker, extended biliary dilatation programs (EBDP). Secondary endpoints included time to csBAS, incidence of bile leak, intervention rates with csBAS, and graft failure. Univariable and multivariable analyses were performed to identify independent associations with csBAS. Graft survival with csBAS was assessed using a Kaplan–Meier curve.

**Results:**

A total of 842 patients underwent 864 LTs with DD anastomosis, of which 123 LTs (14.2%) developed csBAS. The mean age and follow up time were 53.3 ± 11.3 years and 7.0 ± 5.0 years, respectively. Year of transplant (*p* < 0.01), donor age (*p* = 0.01), suture material (*p* = 0.05) and suture technique (*p* = 0.01) were associated with csBAS on univariable analysis. On multivariable analysis, only donor age (adjusted OR 1.01, 95% CI 1.00–1.03, *p* = 0.03) was found to be independently associated, while absorbable suture material, suture technique and year of transplant were not associated. No difference was seen in bile leaks or graft failure with absorbable suture material nor anastomotic technique. No significant association was observed with time to csBAS, nor between csBAS and graft failure.

**Conclusion:**

Variable suture technique and suture material during DD reconstruction are associated with comparable outcomes following LT.

## Graphical abstract

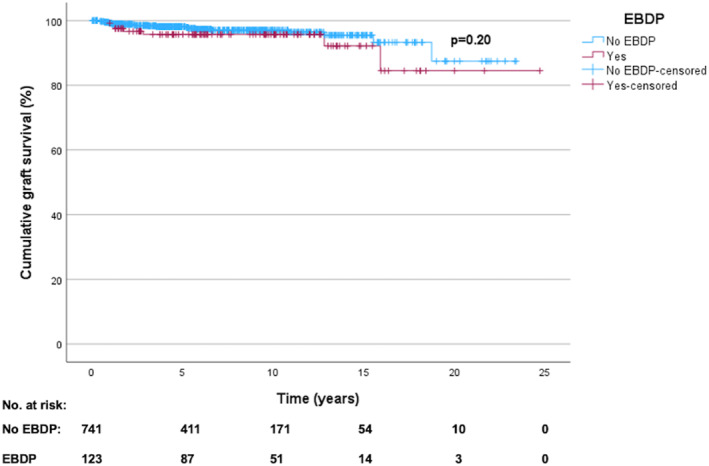

## 1 Introduction

Biliary anastomotic strictures (BAS) complicate 5%–35% of liver transplants (LT) [1, 2]. These can lead to high morbidity, necessitate postoperative interventions, and in some cases, may not be amenable to endoscopic or percutaneous treatment.

Several authors have suggested the role of surgical technique in the development of BAS [3, 4, 5]. Although the type of biliary anastomosis (e.g., duct‐to‐duct [DD] and Roux‐en‐Y hepaticojejunostomy [HJ]) has been widely investigated, there is a paucity of literature evaluating other aspects of the anastomosis such as suture technique and the use of absorbable suture material. The existing literature suggests that non‐absorbable suture material may increase the risk of biliary complications such as strictures and cholangitis while interrupted and continuous anastomotic suturing techniques may have comparable outcomes [3, 6, 7]. This literature comprises predominantly retrospective analyses with patient cohorts undergoing non‐transplant hepatobiliary surgery. However, in our prior study in patients with primary sclerosing cholangitis (PSC) who underwent LT, we highlighted that BAS outcomes can be influenced by the type of anastomosis [8]. This bias arises secondary to conventional BAS definitions (e.g., stricture or abnormality requiring dilatation or stenting) which commonly rely on a single intervention as the defining feature. There is also significant heterogeneity in definitions for BAS used in the literature, conferring further bias. For example: a radiological diagnosis of BAS may be confounded by frequently encountered duct irregularities or mismatch. Lastly, DD anastomosis poses greater accessibility for opportunistic intervention with endoscopic retrograde cholangiopancreatography (ERCP). This is compared to more challenging postoperative access with percutaneous transhepatic cholangiography (PTC) following HJ, which ultimately contributes to artificially inflated BAS rates with DD anastomosis when evaluating surgical technique. This was also corroborated in our study investigating BAS outcomes with transcystic stenting in 677 LTs for various pathologies extending beyond PSC [9].

To overcome the challenges associated with conventional definitions, we previously described a novel definition—extended biliary dilatation programs (EBDP) [8]. This surrogate marker for clinically significant BAS (csBAS) was defined as requiring more than three ERCP or PTC interventions (stenting or dilatation) consecutively. We speculated that EBDP mitigated the influence of opportunistic intervention and center‐specific differences in protocols for early intervention in patients with cholestatic clinical pictures. As study of associations between surgical technique and BAS have likely been confounded by these limitations, further investigation using csBAS—restricted to a single anastomosis—is necessary to facilitate more accurate assessment of risk factors. This study therefore aimed to identify modifiable surgical risk factors by evaluating the influence of suture material and technique on the incidence of csBAS in patients who underwent LT with DD anastomosis at a single center.

## 2 Method

### 2.1 Study Design

A retrospective cohort study was performed reviewing the medical records of all adult patients (≥ 18 years of age) who underwent LT with DD anastomosis at Austin Health in Melbourne, Victoria, Australia between January 1st, 2000 to December 31st, 2023. Cases were excluded from data collection if (a) HJ was performed, (b) multi‐visceral (liver, stomach, pancreas, intestine) transplantation was performed, (c) intraoperative death occurred, (d) death occurred prior to completion of biliary reconstruction (in cases of staged reconstruction), (e) there was evidence of hepatic artery thrombosis postoperatively, (f) sizable cholangiocarcinoma on explant pathology which would have precluded transplantation, or (g) insufficient data was recorded regarding surgical technique (i.e., both suture technique and material were missing). In cases of early re‐transplantation within 90 days, the initial LT was excluded.

### 2.2 Liver Transplantation

The routine piggy‐back technique was used in the majority of LTs. Transplanted livers included either whole or split grafts retrieved from only deceased donors (brain or circulatory death). The type of anastomosis, suturing material and technique, and intraoperative stenting were performed at the surgeon's discretion [10, 11].

Factors which influenced biliary anastomotic technique included (a) recipient disease, (b) preoperative recipient biliary tract imaging (e.g., magnetic resonance [MRCP] or ERCP) and (c) intraoperative evaluation of (i) duct size disparity, (ii) thickness of ducts, and (iii) resistance to passage of the probe through the common bile duct. Anastomotic technique at our institution included either HJ or DD reconstruction. Anastomotic technique was (a) continuous suture, (b) continuous closure of the posterior wall and interrupted anteriorly (herein referred to as the combined technique), or (c) interrupted suture. Notably, the combined technique was initially performed in 2014 and increasingly performed after 2018. Duct sizes were infrequently reported in the record, however, at our institution, continuous suturing is generally preferred with larger sizes (≥ 5 mm) and interrupted closure with smaller duct sizes ≤ 5 mm. Suture material used included absorbable (Maxon, PDS polydioxanone, Vicryl) or non‐absorbable (Prolene polypropelene) suture.

### 2.3 Outcomes

Our primary endpoint was the rate of csBAS evaluated using the surrogate marker, EBDP, as previously defined [8]. While we hypothesize that EBDP increases the specificity for csBAS, it is noted that this definition has not yet been prospectively validated given its recent introduction and, to our knowledge, has only been used at our institution. We also acknowledge that the threshold chosen to meet criteria for EBDP may simultaneously reduce the sensitivity for detecting csBAS.

Secondary endpoints included the time to csBAS (recorded as the time from LT to initial intervention in EBDP), incidence of bile leak, PTC and ERCP intervention rates, and graft failure. Bile leak was defined according to Koch et al. with cystic stump leak following T‐tube removal excluded [12]. Graft failure was defined as requiring re‐transplantation more than 90 days after initial LT with censoring for death. The 90‐day period reduces confounding from causes of early graft loss including primary non‐function and allows adequate time for development of BAS. Associations with mortality were not assessed as these were likely to be confounded by alternative causes or the variable exposure to the risk of death as previously seen [8].

### 2.4 Statistical Analysis

Demographics and clinical characteristics were summarized with descriptive statistics. Intraoperative data included suturing technique and material, intraoperative stenting, use of normothermic machine perfusion (NMP), donation pathway (donation after brain death vs. circulatory death), warm ischemic time and cold ischemic time. Warm ischemic time was defined as the duration from allograft removal from ice to portal venous unclamping. Cold ischemic time was defined as the duration from allograft placement onto ice following explantation to removal from ice for allografts under static cold storage. Duration of NMP was excluded from cold ischemic time where ex vivo perfusion was utilized. Hepatic artery delay was defined as the time from portal vein reperfusion to hepatic artery reperfusion. Postoperative data included rates of csBAS, bile leaks, interventions with csBAS, time to strictures and graft failure.

Continuous variables underwent normality assessment using the Kolmogorov–Smirnov test and were presented as mean and standard deviation (SD) (if normally distributed) or median and interquartile range (IQR; if not normally distributed). Continuous variables were compared using an independent *t*‐test (if normally distributed) or Mann Whitney *U*‐test (if not normally distributed). A univariable analysis using Chi‐Square and logistic regression (for categorical and continuous variables respectively) was performed to identify covariates for multivariable analysis. Binomial logistic regression was subsequently conducted with inclusion of all covariates with *p* < 0.10 on univariable analysis to identify independent associations with csBAS. Temporal subgroup analysis with transplant era (pre‐ and post‐2012) was not performed due to the low csBAS rate with absorbable suture material. It was hypothesized that such subgroup analysis would be underpowered and risk statistical validity. Furthermore, such analysis would not accurately assess the effect of suture technique (particularly the combined technique due to its performance in the latter half of the study period) and therefore further risk temporal confounding.

Time to graft failure in patients who developed csBAS was assessed using a Kaplan–Meier curve with log‐rank test for comparison. All statistical tests were two‐sided; *p* < 0.05 was considered significant. Statistical analysis was performed using IBM SPSS v29 (New York, United States).

## 3 Results

Overall, 842 patients underwent a total of 864 LTs with DD anastomosis. Of these, 788 (91.2%) were whole graft, 47 (5.4%) were split grafts, and in 29 (3.3%) graft nature was not documented (notably in the early study period). The mean age and follow up time were 53.3 ± 11.3 years and 7.0 ± 5.0 years, respectively. Perioperative characteristics of LTs with DD anastomosis are reported in Table 1.

**TABLE 1:** Demographics and perioperative clinical characteristics in liver transplants with duct‐to‐duct biliary reconstruction.

Variable		Mean (SD)	Count (%)
Recipient age (years)		53.3 (11.3)	
Recipient sex: male			576 (66.7)
Donor age (years; missing: 4)		46.1 (16.5)	
Donor sex: male			471 (54.5)
Indication: Biliary obstructive cause			104 (12.0)
Suture material	Absorbable		76 (8.8)
	Non‐absorbable		788 (91.2)
Suture technique	Continuous and interrupted		210 (24.3)
	Continuous		420 (48.6)
	Interrupted		212 (24.5)
	Missing		22 (2.5)
Intraoperative stent	No stent		476 (54.1)
	Stented		366 (42.4)
	Missing		22 (2.5)
Normothermic machine perfusion			31 (3.6)
Hepatic artery reperfusion delay (mins)		61 (46)	
Cold ischemic time (mins)		398 (126)	
Warm ischemic time (mins)		46 (11)	

*Note:* Biliary obstructive cause (Indication) included primary sclerosing cholangitis, primary biliary cirrhosis, ischemic cholangiopathy from prior transplantation.

### 3.1 Clinically Significant Stricture Rates

A total of 123 LTs (14.2%) developed csBAS while 286 LTs (33.1%) developed BAS using conventional definitions.

Variables associated with csBAS on univariable analysis (Tables 2 and 3) included year of transplant (*p* < 0.01), donor age (*p* = 0.01) and suture technique (*p* = 0.01). No statistically significant association was identified between suture material and csBAS (*p* = 0.05). As seen in Table 4, only donor age (adjusted OR 1.01, 95% CI 1.00–1.03, *p* = 0.03) was found to be independently associated with csBAS on multivariable analysis.

**TABLE 2:** Evaluation of clinically significant biliary anastomotic strictures with absorbable suture materials and variable suture techniques in liver transplants with duct‐to‐duct biliary reconstruction.

Variable		Total	EBDP	No EBDP	OR [95% CI]	*p* value
Suture material	Absorbable	76 (8.8)	5 (6.6)	71 (93.4)	0.40 [0.16,1.01]	0.05
	Non‐absorbable	788 (91.2)	118 (15.0)	670 (85.0)		
Suture technique	Continuous and interrupted	210 (24.3)	16 (7.6)	194 (92.4)		0.01
	Continuous	420 (48.6)	72 (17.1)	348 (82.9)		
	Interrupted	212 (24.5)	33 (15.6)	179 (84.4)		
	Missing	22 (2.5)	2 (9.1)	20 (90.9)		

Abbreviations: EBDP, extended biliary dilatation program.

**TABLE 3:** Univariable analysis assessing associations with clinically significant biliary anastomotic stricture in liver transplants with duct‐to‐duct biliary reconstruction.

Variable	*p* value
Hepatic artery reperfusion delay	0.86
Donation pathway	0.80
Donor age	0.01
Donor sex	0.34
Indication: Biliary obstructive cause	0.65
Intraoperative stenting	0.94
Normothermic machine perfusion	0.76
Postoperative bile leak	0.77
Recipient age	0.20
Recipient sex	0.38
Suture material	0.05
Suture technique	0.01
Cold ischemic time	0.68
Warm ischemic time	0.22
Year of transplant	< 0.01

**TABLE 4:** Multivariable analysis assessing associations with clinically significant biliary anastomotic strictures in liver transplants with duct‐to‐duct biliary reconstruction.

Covariate		Adjusted OR [95% CI]	*p* value
Donor age		1.01 [1.00–1.03]	0.03
Absorbable suture material		0.45 [0.14–1.51]	0.20
Suture technique	Total		0.25
	Combined versus continuous	0.58 [0.30–1.10]	0.10
	Combined versus interrupted	0.61 [0.30–1.24]	0.17
	Interrupted versus continuous	1.05 [0.67–1.67]	0.82
Year of transplant		0.96 [0.92–1.01]	0.08

Mean times to csBAS with the combined, continuous, and interrupted techniques were 8.2 ± 12.1, 11.8 ± 21.9, and 6.3 ± 6.5 months, respectively, with no difference observed in comparisons between groups (*p* > 0.05). The mean times to csBAS with absorbable and non‐absorbable sutures were 6.6 ± 7.5 and 10.0 ± 18.1 months, respectively (*p* = 0.68).

### 3.2 Bile Leak

Postoperative bile leaks were encountered in 17 LTs (2.0%). No difference was seen with different suture technique (combined 4/210 [1.9%] vs. continuous 6/420 [1.4%] vs. interrupted 7/212 [3.3%], *p* = 0.28) nor absorbable suture material (non‐absorbable 17/788 [2.2%] vs. absorbable 0/76 [0%], *p* = 0.20).

### 3.3 Intervention Rates

The mean number of interventions in LTs complicated by csBAS was 6.2 ± 2.6 (ERCP 5.9 ± 2.5, PTC 0.3 ± 1.1).

### 3.4 Death‐Censored Graft Failure

Twenty‐seven LTs were complicated by graft failure. Graft failure was due to biliary pathology (*n* = 13, including ischemic cholangiopathy, recurrent biliary cirrhosis, PSC and secondary biliary cholangitis), viral hepatitis (*n* = 5), rejection (*n* = 5), primary poor function (*n* = 1), recurrent metabolic disease (*n* = 1), nodular regenerative hyperplasia (*n* = 1), and acute hepatic necrosis (*n* = 1).

**FIGURE 1:**
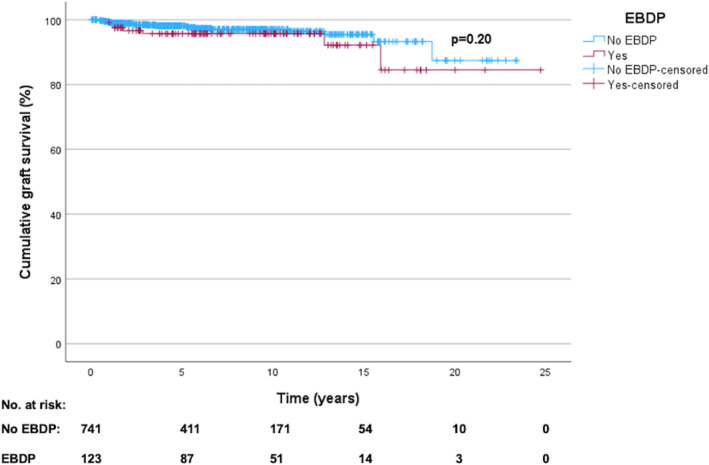
Cumulative graft survival in liver transplants with duct‐to‐duct anastomosis with and without clinically significant biliary anastomotic strictures. Clinically significant biliary anastomotic strictures represented using surrogate marker, extended biliary dilatation programs (EBDP). Data presented using a Kaplan–Meier curve with log‐rank test performed. Figure created using IBM SPSS v29 and Microsoft Word.

No statistically significant association was found between csBAS and death‐censored graft failure (csBAS 5.7% [7/123] vs. no csBAS 21/747 [2.8%], OR 2.09, 95%CI 0.87–5.02, *p* = 0.09). Cumulative graft survival was comparable with and without csBAS (*p* = 0.20) (Figure 1).

## 4 Discussion

In recent decades, DD anastomosis has become more widely accepted as the preferred biliary reconstructive technique during LT when compared to HJ. This has been supported by non‐inferiority in biliary complications, lower surgical complexity, and preservation of native duct anatomy reducing the likelihood of recurrent ascending cholangitis while enabling endoscopic access for postoperative biliary intervention [8].

Although researchers have sought to compare different methods of biliary anastomosis with respect to BAS incidence, limitations in conventional definitions for BAS challenge the accuracy of these findings. In particular, we note that minor liver function test derangements may prompt biliary imaging which can identify insignificant findings such as duct mismatch querying a stricture. This may be opportunistically treated with dilatation or stenting during ERCP. We therefore recently described the definition, EBDP, to help negate the impact of these opportunistic interventions on inflated BAS rates [8]. We speculate that these anastomoses are inherently incomparable using conventional definitions and that the use of EBDP as a surrogate marker provides greater specificity for csBAS, particularly with DD anastomosis.

In our retrospective 23‐year cohort analysis, we used EBDP as a surrogate marker to investigate the role of biliary anastomotic technique on the development of csBAS in LTs with DD anastomosis.

We found no reliable association between csBAS and suture technique. The current study corroborated the literature which found no difference in BAS with DD anastomosis using a continuous versus interrupted biliary anastomotic technique [7, 13]. This is despite proposed pathophysiological benefits which include improving microvascular perfusion with interrupted suturing (limiting pursestring effect) or preventing bile leak with continuous suturing [14]. We also investigated a combined technique that has recently (since 2018) been commonly used for convenience when access to the posterior wall for interrupted closure was challenging. While an association between suture technique and csBAS was observed on univariable analysis, an independent association was not identified when accounting for confounding (e.g., year of transplant). Given the 23‐year study period, we speculated that the relationship seen on univariable analysis was likely confounded by the recency of increasing use of the combined technique. This is also relevant when considering advancements in post‐transplant care over the study period. Our findings corroborated a retrospective cohort analysis of 394 LTs (287 DD)—where no difference in the rate of BAS between continuous and continuous plus interrupted suturing for biliary anastomoses was identified [15].

We also found no independent association between csBAS and suture material. Although the multivariate model was adequately fitted, a low event rate of csBAS (five cases) was observed with absorbable suture material—likely partially attributable to the preference toward non‐absorbable suture material use observed. Therefore, while the overall model was stable, the adjusted effect of suture material can carry more uncertainty. Despite this limitation, our findings corroborated a prior retrospective cohort analysis of 509 LTs (476 DD, 33 HJ) which used a biochemical and radiological definition for BAS. Although nonabsorbable suture material was associated with greater odds of overall anastomotic complications (OR 2.45, 95% CI 1.09–5.54, *p* = 0.03), the authors found no independent association with BAS on multivariable analysis (*p* = 0.09) [3]. In future, a prospective randomized trial to increase the representation of absorbable suture material use may be performed to more precisely evaluate the relationship between suture material and csBAS.

Several studies (evaluating mixed cohorts with DD and HJ) have implicated donor age as an independent risk factor in the development of non‐anastomotic biliary strictures [16, 17, 18, 19]. However, its role as a risk factor for BAS remains controversial [3, 9, 20, 21]. Although our findings indicated an independent association with csBAS, it is acknowledged that its influence may not be clinically substantial (adjusted OR 1.01, 95% CI 1.00–1.03).

We found no association between csBAS and previously proposed risk factors including intraoperative stenting, postoperative bile leak, recipient sex and age, donor sex, NMP use, donation pathway, and ischemic times [9, 22, 23]. No differences were seen in rates of bile leak and graft failure with variable suture techniques nor absorbable material. Additionally, we found no association between csBAS and graft failure which supported the literature despite the limitations of conventional definitions [22]. We speculated that the availability and accessibility to treatments for csBAS with DD anastomosis may mitigate the risk of developing graft failure.

We acknowledge several limitations. Firstly, this study was performed at a single institution which carried inherent selection bias affecting the replicability of these results. There is also a risk of temporal bias as the study spanned 2 decades during which there have been significant improvements in LT practices related to surgical and post‐transplant care. For example: NMP was recently implemented in 2017 on a case‐by‐case basis and the combined suture technique was performed since 2014. Intraoperative biliary stenting was also less commonly performed in the latter half of the study period after no significant benefit was identified in routine performance [9]. Additionally, as the widely accepted definition for bile leak was introduced in 2011, grade A bile leak may be missed if drain tube bilirubin levels were not performed [12]. Thirdly, data was collected retrospectively and therefore relied heavily upon the accuracy of medical documentation. As such, decision‐making surrounding the choice of technique cannot be accurately inferred. Furthermore, as EBDP has (to our knowledge) only been utilized at our institution, heterogeneity in BAS definitions affect the replicability of these results and comparisons with the literature. We also acknowledge that the presence of concurrent non‐anastomotic strictures may influence the results with regards to intervention rates. As the aim of this study was to identify modifiable surgical risk factors for the development of csBAS, non‐anastomotic strictures—which arise predominantly due to ischemic or immune‐mediated injury rather than surgical technique—were not evaluated in this study [24].

Although it is widely theorized that surgical technique plays a key role in the development of BAS, there is a paucity of high‐quality literature investigating aspects of surgical technique in the transplant setting. Existing literature is predominantly retrospective and includes mixed cohorts with different anastomoses—the latter which we speculated confers inherent bias (surgeons are more prone to adopt running sutures in larger ducts and interrupted sutures in small ones). This highlights the need for using standardized definitions for identifying csBAS across different anastomotic techniques. In the interim, we recommend that cohort studies focusing on aspects of surgical technique should restrict analysis to a single anastomosis (e.g., evaluating DD or HJ individually rather than using mixed cohorts).

## 5 Conclusion

Variable suture technique and suture material during DD reconstruction are associated with comparable outcomes following LT.

## Author Contributions

**Samith Minu Alwis:** conceptualization, data curation, formal analysis, methodology, software, writing – original draft, writing – review and editing. **Robert Torode:** data curation, methodology, writing – review and editing. **Michael Anthony Fink:** writing – review and editing. **Ruelan Furtado:** writing – review and editing. **Eunice Lee:** writing – review and editing. **Graham Starkey:** writing – review and editing. **Robert Jones:** writing – review and editing. **Marcos Vinicius Perini:** conceptualization, data curation, methodology, project administration, supervision, validation, writing – original draft, writing – review and editing.

## Ethics Statement

This study was performed in line with the principles of the Declarations of Helsinki and Istanbul and in accordance with the National Health and Medical Research Council (NHMRC) National Statement on Ethical Conduct in Human Research (2007) and the principles of Good Clinical Practice. Ethical approval for the study was provided by Austin Health Human Research and Ethics Committee (#23113).

## Conflicts of Interest

The authors declare no conflicts of interest.

## Data Availability

The data that support the findings of this study are available on request from the corresponding author. The data are not publicly available due to privacy or ethical restrictions.
